# The Role of mTOR Inhibitors in Hematologic Disease: From Bench to Bedside

**DOI:** 10.3389/fonc.2020.611690

**Published:** 2021-01-08

**Authors:** Yimei Feng, Xiaoli Chen, Kaniel Cassady, Zhongmin Zou, Shijie Yang, Zheng Wang, Xi Zhang

**Affiliations:** ^1^Medical Center of Hematology, The Xinqiao Hospital of Third Military Medical University, Chongqing, China; ^2^State Key Laboratory of Trauma, Burns and Combined Injury, Third Military Medical University, Chongqing, China; ^3^Chongqing Sub-center of National Clinical Research Center for Hematologic Disease, Chongqing, China; ^4^Irell and Manella Graduate School of Biological Sciences of City of Hope, Duarte, CA, United States; ^5^Department of Chemical Defense Medicine, School of Military Preventive Medicine, Third Military Medical University, Chongqing, China

**Keywords:** rapamycin, Rapalogs, mTOR pathway, malignant and benign hemopathies, graft-versus-host disease

## Abstract

The mTOR pathway plays a central role in many cellular processes, such as cellular growth, protein synthesis, glucose, and lipid metabolism. Aberrant regulation of mTOR is a hallmark of many cancers, including hematological malignancies. mTOR inhibitors, such as Rapamycin and Rapamycin analogs (Rapalogs), have become a promising class of agents to treat malignant blood diseases—either alone or in combination with other treatment regimens. This review highlights experimental evidence underlying the molecular mechanisms of mTOR inhibitors and summarizes their evolving role in the treatment of hematologic disease, including leukemia, lymphoma, myeloma, immune hemocytopenia, and graft-versus-host disease (GVHD). Based on data presented in this review, we believe that mTOR inhibitors are becoming a trusted therapeutic in the clinical hematologist’s toolbelt and should be considered more routinely in combination therapy for the management of hematologic disease.

The mammalian target of rapamycin (mTOR) is a downstream target of multiple signaling pathways involved in biological activities, including the familiar PI3K/Akt/mTOR signaling pathway, which plays an important role in cell growth, differentiation, metastasis and survival, and has become an important target of cancer treatment. Rapamycin (Rapa), the first mTOR inhibitor, was initially used as an immunosuppressive drug in the field of solid organ transplantation (1). In-depth studies of rapamycin and its analogs (Rapalogs), and of the mTOR signaling pathway, have led to the understanding that Rapalogs can not only induce tumor cell apoptosis, cell cycle arrest and signal transduction inhibition, but that they also affect gene transcription and epigenetic regulation. The mTOR signaling pathway is critical in normal myeloid and lymphoid development and function. Moreover, hyperactivation of mTOR is a hallmark of many hematological diseases and provides a strong rationale for the use of mTOR inhibitors (mTORi), such as Rapalogs. The primary focus of this review is to 1) review the literature on the regulation and outcome of the mTOR signaling pathways from a basic research perspective, and 2) summarize the use of mTOR inhibitors to target aberrant mTOR activation and signaling in hematological diseases, such as acute leukemia, Hodgkin lymphoma, non-Hodgkin lymphoma, multiple myeloma, GVHD, and Waldenström macroglobulinemia. We also provide our perspective on the evolution of this field, as the application and efficacy of mTOR inhibitors becomes more widely recognized in the field of blood disorders.

## mTOR And mTOR Inhibitors

The mammalian target of rapamycin (mTOR) is a large (289 kDa), conserved serine protein kinase, belonging to the PI3K kinase family. mTOR can integrate the stimulatory signals from nutrients, growth factors and environmental pressure, and regulate cell growth, proliferation, differentiation, and cell cycle progression. In the immune system, mTOR can transmit and integrate signals from the immune microenvironment, and is considered a key regulator of immune metabolism and function (2). At present, it is believed that there are two dominant, upstream signaling pathways for mTOR, one is the PI3K/Akt/mTOR pathway, which interacts with mTOR to positively regulate, and the other is the LKB1/AMPK/mTOR pathway, which has primarily been reported as a negative regulator of mTOR (3, 4).

**Figure 1:**
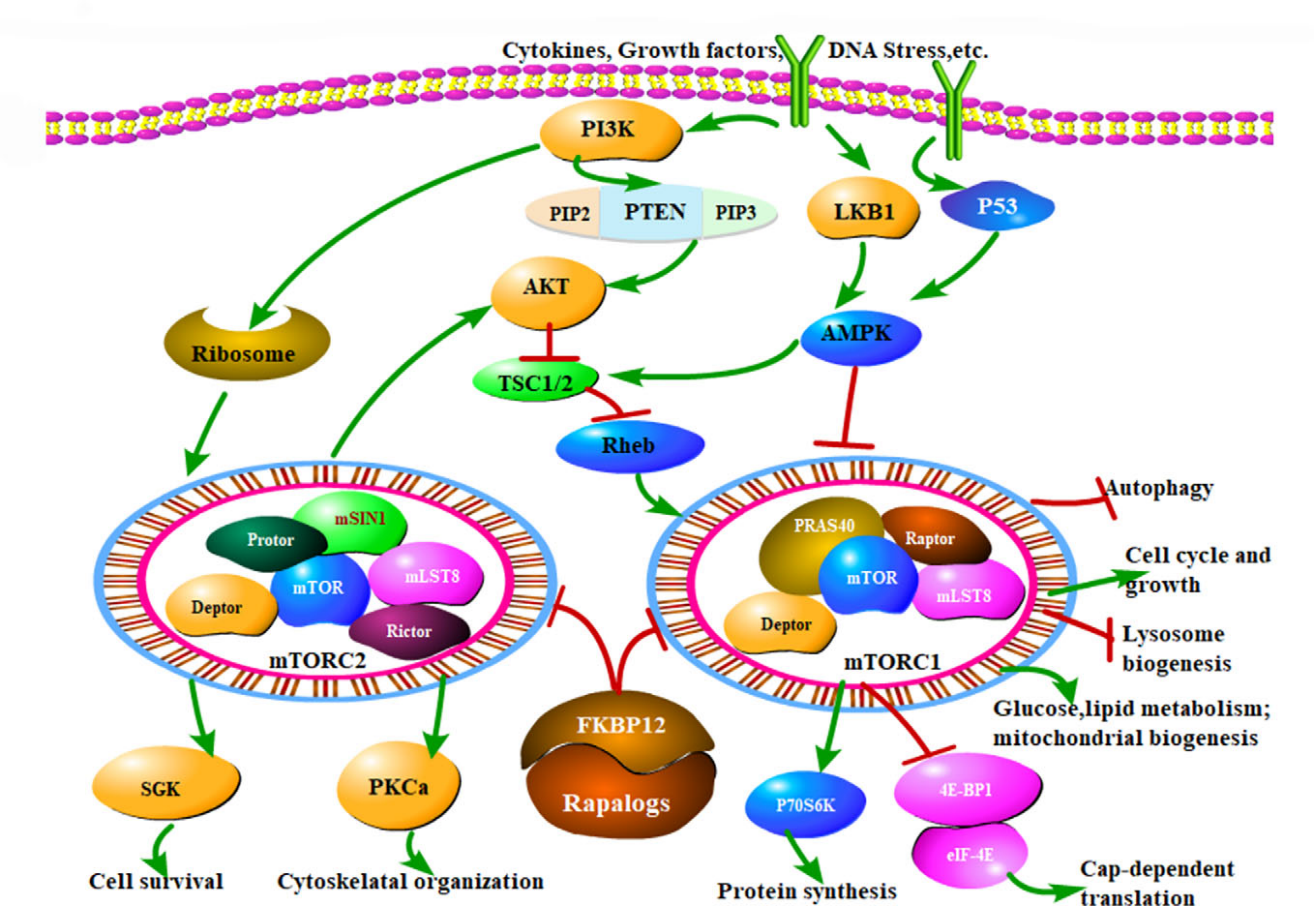
The mTOR signaling pathway and Rapalog therapeutic mechanisms. mTOR modulates a variety of cellular activities through mTORC1 and mTORC2. MTOR can respond to extracellular stimuli, such as cytokines, growth factors and DNA stress, which mainly regulate cell growth, cell cycle, and other physiological activities through PI3K/Akt/mTOR pathway. Normally, TSC1/TSC2 forms a dimer complex, which is an inhibitor of small GTPase Rheb, and Rheb is a necessary stimulating protein for mTOR activation. Hence the mTORC1 activity is critically inhibited by TSC1/TSC2 complex. When Akt is activated, it can phosphorylate ser939 and thr1462 of TSC2 and inhibit the formation of TSC1/TSC2 complex, thus relieving the inhibition of Rheb, then activated Rheb sensitized mTOR function. Additionally, PI3K activates the ribosome to increase mTORC2 reaction. In a word, PI3K promotes the mTOR activates. Another important pathway of mTOR is regulated by AMPK. Firstly, AMPK directly phosphorylates Raptor to inhibit mTORC1 activity. Raptor is the downstream molecule of AMPK, and TSC2 is not involved in this process. Secondly, AMPK can also activate TSC1/TSC2 complex and inhibit mTORC1 activity as described above. Therefore, unlike the PI3K, AMPK restrains the mTOR function. Rapalogs can combine the FKBP12 and inhibit the kinase activity of mTORC1 and mTORC2, ultimately regulate various biological functions of cells, that is the mechanism of mTOR inhibitors. *Arrows indicate activation and bars represent inhibition*.

mTOR exerts its activity *via* the formation of two catalytically distinct complexes, mTORC1 and mTORC2 (5). mTORC1 complex is composed of mLST8, PRAS40, Deptor, and Raptor. mTORC2 complex is composed of Rictor, mSIN1, Protor, mLST8, and Deptor. In general, mTORC1 plays a more dominant role in regulating cellular anabolic processes, while the effect of the mTORC2 signaling pathway is primarily through Akt Studies have shown that mTORC1 signaling pathway plays an important role in the metabolism of glucose, lipids, nucleotides, and proteins as well as in the regulation of mitochondrial biogenesis (6). The downstream signaling molecules of mTORC1 are primarily P70S6K (p70 ribosomal S6 kinase) and 4E-BP1 (eukaryotic transcription initiation factor binding protein), by which protein translation and synthesis are increased (**Figure 1**). On the other hand, mTORC2 is primarily involved in the insulin signaling pathway to regulate glucose and lipid metabolism (7). Importantly, when mTORC1 is completely inhibited by mTOR inhibitors, mTORC2 signaling is preserved (8).

Several pharmacological agents have been developed which inhibit mTOR (mTOR inhibitors; referred to broadly here as mTORi), including Rapamycin and its analogs. Rapamycin, clinically named sirolimus (SRL), was a macrolide antibiotic discovered in the 1970s (9). SRL has many functional properties, including inhibition of yeast growth, anti-cancer, anti-aging and anti-atherosclerotic effects as well as immune regulation (10–12). In the context of immune regulation, SRL can inhibit T cell activation and proliferation, and restrain B cell activation and antibody production caused by antigens and cytokines (13). In the clinic, SRL is primarily used in the prevention of organ rejection after solid organ transplantation, as well as for the treatment of autoimmune diseases (14). With the development of Rapamycin analogs in recent years, Rapalogs, many studies have reported their therapeutic effects on hematological diseases (15).

**Figure 2:**
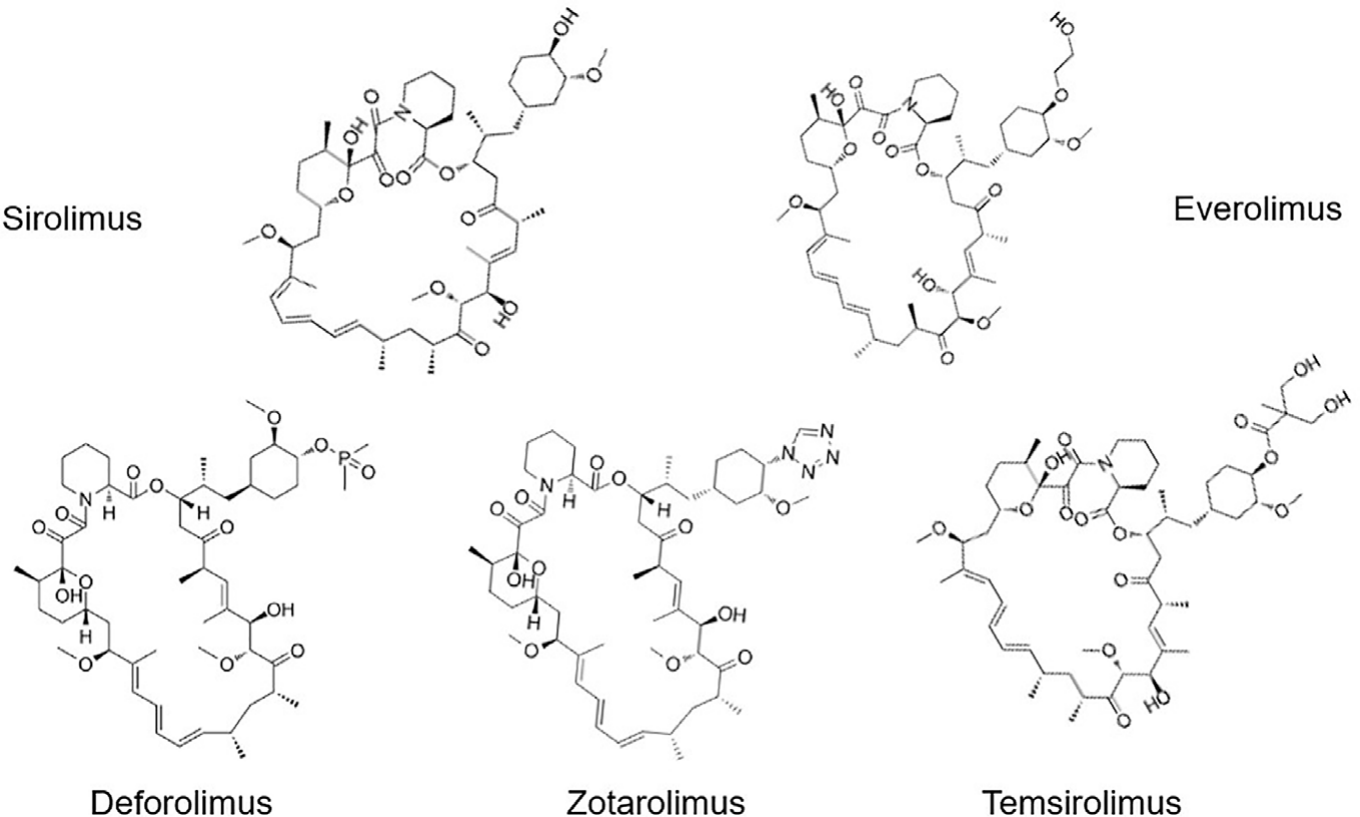
Chemical structure of five mTOR inhibitors.

To date, the most common Rapalogs are: Everolimus (Eve, RAD-001), Deforolimus (Def, Ridaforolimus), Zotarolimus (Zot), and Temsirolimus (Tem, CCI-779) (**Figure 2**). Everolimus is an mTOR inhibitor developed by Novartis (Switzerland), which is a 40-o-(2-hydroxyethyl) derivative of rapamycin, with the molecular formula C_53_H_83_NO_14_, (958 kDa). In March 2009, the drug received accelerated approval from the FDA for the treatment of kidney cancer (16, 17). Deforolimus is a C40 derivative of Rapa, which is a semi-synthetic derivative designed by Computer-aided drug design (CADD) with molecular formula C_53_H_84_NO_14_P (990 kDa) (18). Def is still considered an investigational agent in the management of advanced solid tumors, such as sarcoma (19) and breast cancer (20). Only clinical study reported using Def for the treatment of the hematological malignancy lymphoma, and the overall response rate (ORR) was 50% (21). Zotarolimus is a Rapa derivative developed by Abbott and Medtronic. It is a C40 tetrazole substitute of Rapa, with molecular formula C_52_H_79_N_5_O_12_ (966 kDa). Compared with Rapa, Zot has a shorter half-life *in vivo*. The mechanism of Zot is similar to Rapa. Zot combines with FKBP12 to form a complex, which bind to mTOR protein kinase to form a trimer, inhibits the activity of mTOR by preventing its phosphorylation, and prevents cell cycle progression from G1 to S phase (22). It was designed for use as a coating in stents with phosphorylcholine as a carrier. Temsirolimus, also named 42-[3-hydroxy-2- (hydroxymethyl)-2-methylpropionate], is a Rapa derivative with molecular formula C_56_H_87_NO_16_ (1030 kDa). It is a Rapa soluble esterified derivative officially approved by the US FDA in 2007, which inhibits the translation of several key proteins for cell cycle regulation. These effects lead to cell cycle arrest in the G1 phase, therefore also known as Cell Cycle Inhibitor-779 (CCI-779). In August 2007, FDA approved temsirolimus as a first-line treatment for advanced renal cancer. At present, it is marketed as a treatment of renal cancer, and is being investigated in the clinic for the treatment of breast cancer (23), lymphoma (24), lung cancer (25), and many other tumors (26).

## Applications of mTORi in Hematological Malignancies

### Acute Leukemia

Acute myeloid leukemia (AML) is a type of cancer of the bone marrow in which myeloid stem cells differentiate and accumulate as abnormal myeloblasts in the bone marrow, impair erythropoiesis/hematopoiesis and eventually migrate throughout the body. Although mTOR activation is frequently observed in AML blasts, the precise function and the downstream targets of mTOR in this disease are poorly understood. Feng et al. revealed that PFKFB3 was a novel downstream substrate of mTOR signaling pathway, and found that up-regulation of PFKFB3 *via* aberrant mTOR signaling was essential for AML cell survival. Moreover, PFKFB3 inhibitor PFK15 and rapamycin synergistically ablated AML cell proliferation (THP1 and OCI-AML3 cells) (27). Xu et al. found that PI3K/Akt/mTOR pathway was up-regulated in most AML cells. Rapa combined with etoposide, a chemotherapy, enhanced the effect of etoposide on primary AML cells; moreover, leukemia progenitor cells were more sensitive to Rapa than normal progenitor cells (28, 29). Brown et al. have found that rapamycin inhibited growth of B-precursor ALL cell lines *in vitro*, as evidenced by apoptotic cell death. Mice with advanced ALL and were treated with rapamycin as a single agent exhibited enhanced survival compared to control-treated ALL-bearing animals (30). Avellino et al. reported SRL (+/- doxorubicin) can promote the apoptosis of primary pediatric ALL blast cells *in vitro*, indicating that rapamycin targets two pathways that are crucial for cell survival and chemoresistance of malignant lymphoblasts (PI3k/Akt and FKBP51/NF-kB) (31). In a phase I/II study of everolimus in combination with HyperCVAD in patients with R/R ALL, ORR was in 41% and significantly higher in first salvage patients as compared with second salvage or beyond. Moreover the inhibition of phosphorylation of S6K1 did not correlate with response, and the combination regimen did not increased toxicity than HyperCVAD alone (32). Thus, the combination of Rapalogs with some chemotherapies may be an effective regimen for AML and ALL.

Results of clinical trials using Rapalogs as a single agent have shown very limited response in AML patients, such as sirolimus (33), deforolimus (21), or everolimus (34). There are also reports of combination strategies; for example, SRL was combined with MEC (mitoxantrone, etoposide, cytarabine) to treat patients with relapsed, refractory, or untreated high-risk AML. The ORR to the combination regimen was 16%–47% (35–37), which suggested SRL and MEC may be an effective option as a treatment regimen for AML. However, Burnett et al. added everolimus to consolidation therapy in AML, and reported there was no difference in relapse-free survival and overall survival (38). This study suggests that the addition of everolimus to chemotherapy provides no benefit (38). Interestingly, Recher et al. found that some primary AML cells have innate Rapa-resistance, due to several mechanisms, including FKBP-12 mutations, high eIF-4/4E-BP1 ratio, defective regulation of p27kip1, and c-myc amplification (33). Taken together, combination of Rapalogs with other chemotherapies might be an effective regimen for patients with high-risk acute leukemia; however, stratification of patients who are likely to respond to these regimens, perhaps based on underlying mutation patterns, must be better understood.

### Myelodysplastic Syndrome

Myelodysplastic syndrome (MDS) is a group of hematopoietic stem cell disorders, characterized by ineffective clonal hematopoiesis leading to blood cytopenias, and convey a variable risk of progression to AML (39). Activation of the mTOR pathway in CD33^+^ cells from MDS patients by the amino acid L-leucine in 5q- syndrome was reported by Yip BH et al. (40). Importantly, in primary cells from high-risk MDS patients, not only was mTOR activated but also its downstream targets, P70S6K and 4E-BP1. Treatment with Rapa significantly increased CD33^+^ cells apoptosis from high-risk MDS patients, but not cells from healthy donors or those patients with low-risk MDS (41). Mutated *GSTT1* gene was detected in some MDS patients and this mutation creates a sequence that is 63% homologous to human FKBP-rapamycin associated protein (FRAP). To examine the effect of mutant GSTT-1, two cell lines (K562 and HL-60) were stably transfected with the mutant type *GSTT1* gene. Expectedly, rapamycin induced significant growth inhibition of those two cell lines, suggesting that rapamycin could be included as a potential therapeutic modality for high-risk MDS patients (42).

Platzbecker et al. (43) treated 19 MDS patients with SRL as a single agent, and demonstrated that SRL might have some activity in patients with more advanced MDS but lacked efficacy in low-risk MDS patients. Martin et al. (44) treated 20 MDS patients with temsirolimus at a weekly dose of 25 mg; however, only four (20%) reached the response assessment after 4 months without hematological improvement. Based on these this response assessment, they concluded that Temsirolimus has no beneficial effects in elderly MDS patients. However, because MDS is a highly heterogeneous disease, a more in-depth study on the therapeutic effect of Rapalogs in patients with MDS is warranted.

### Chronic Myeloid Leukemia

CML is a hematopoietic disorder characterized by the malignant expansion of bone marrow stem cells, with the presence of a reciprocal translocation between chromosomes 9 and 22 resulting in activation of fusion gene BCR-ABL expression (45). It has been found that BCR/ABL, a common driver mutation in CML, mediates the expression of VEGF and its transcriptional activator HIF1 through PI3K and mTOR pathways (46). It also found that BCR/ABL regulates P70S6K and 4E-BP1 through mTOR activation (47–49). *In vitro*, Rapamycin (combined with celecoxib) induced cell cycle arrest and apoptosis of the CML cell line K562. As we know, tyrosine kinase inhibitors (TKIs) are the first line therapy for CML, implying that a combination of TKI with mTORi could enhance the antitumor effects of TKI treatment on CML cells (50). Several groups have, indeed, demonstrated this synergistic effect, combining imatinib with rapamycin (47, 51), or everolimus (52) to overcome TKI resistance *in vitro*, including cell line K562, murine CML model and CML patients samples. However, whether Rapalogs represent a new treatment opportunity for TKI resistant CML remains to be investigated in the clinic.

### Myeloproliferative Neoplasms

MPNs often begin with an abnormal gene mutation or change in a stem cell in the bone marrow, resulting in hemocytopenia and splenomegaly, etc. Types of MPN include myelofibrosis, polycythemia vera and essential thrombocythemia (53). Bartalucci et al. found that mTOR signaling pathway was activated in MPN including myelofibrosis (MF), which was located downstream of JAK2 signaling pathway (54). Guglielmelli et al. (55) carried out treatment of patients with myelofibrosis with everolimus, resulting in 20% of patients’ spleens shrinking > 50%, 40% of patients’ spleens shrinking > 30%, and 15%–25% of patients with hematological reaction. In other studies (56, 57), JAK2/V617F mutated leukemia cell lines (HEL and SET2) and patients’ samples of PV or PMF were sensitive to everolimus. Moreover, the combination of JAKs inhibitor (ruxolitinib) and everolimus, showed synergy in inducing cell-cycle arrest and blockade of cell proliferation (58), which is promising for MPN treatment.

### Hodgkin Lymphoma

Aberrant activation of the PI3K/Akt/mTOR pathway is a hallmark of lymphomas, including Hodgkin lymphoma (HL) (59, 60) and non-Hodgkin lymphomas (NHLs) (61, 62). Abnormal activation of upstream or downstream molecules of mTOR can cause disease development. mTOR mutations have been described in diffuse large B-cell lymphoma (DLBCL) samples (63), and activated ABC-DLBCL cell lines expressed high level of S6K1, which is a downstream target of mTOR (64). mTOR directly mediates Cyclin D1 downregulation through glycogen synthase kinase (GSK)-3b in mantle cell lymphoma (MCL) (65). Based on these observations and preclinical data, clinical trial using Rapalogs have been carried out in patients with lymphoma (66).

Everolimus has significant activity in relapsed/refractory (R/R) HL and is currently listed as a treatment option for HL in the National Comprehensive Cancer Network (NCCN) guidelines (67). Everolimus demonstrates single-agent activity in treating Hodgkin (67–71) and non-Hodgkin lymphoma (72–76), with responses ranging from 20% to 47%. In a small phase 2 study, 19 patients with R/R HL were treated with single-agent everolimus (10 mg/d), with an ORR of 47% (69). This was recapitulated in a larger multicenter study of 57 patients with an ORR of 42%, including five complete responses (CRs) and a median PFS of 9 months (68). The combination of sirolimus and HDAC inhibitor (vorinostat) is well-tolerated with encouraging activity in very heavily pretreated patients with Hodgkin lymphoma who are refractory to standard therapies. In 28 patients enrolled and accepted SRL and vorinostat scheme, the ORR was 57% (CR 32%+PR 25%) (77, 78). The emergence of effective new drugs in the treatment HL, including CD30 mAbs and checkpoint inhibitors, such as PD-(L)1 mAbs, may limit the broad application of mTORi in this setting. However, mTORi may be useful in later lines of therapy for difficult-to-treat, multidrug-exposed patients with R/R HL.

### Non-Hodgkin Lymphomas

There are also clinical trials using Rapalogs to treat NHL, for example, temsirolimus is used for relapsed MCL with an ORR 38-47% (79, 80). A phase I b clinical trial investigated temsirolimus in association with R-CHOP (R-CHOP-T), or high-dose cytarabine plus rituximab (R-DHA-T), or fludarabine, cyclophosphamide plus rituximab (R-FC-T). Regarding efficacy, ORR during treatment period was 40% for R-CHOP-T including two patients who reached CR, 43% for R-FC-T including three patients who reached CR, and 47% for R-DHA-T including six patients who reached CR (81). In another study, Temsirolimus combined with bortezomib in DLBCL achieved an ORR of 31% (82). Yet another study investigated single-agent temsirolimus 25 mg weekly administrated in three groups of patients with relapsed aggressive and indolent lymphomas: group A (diffuse large B-cell lymphoma, transformed follicular lymphoma), group B (follicular lymphoma), and group C (chronic lymphocytic leukemia/small lymphocytic lymphoma, and other indolent lymphomas). Group A had an overall and complete response rate of 28.1% and 12.5%, respectively, and median PFS of 2.6 months and median OS of 7.2 months. Group B had overall and complete response rates of 53.8% and 25.6%, respectively, and median PFS of 12.7 months; median OS has not yet been reached. Group C had a partial response rate of 11% with no complete responders (83). Everolimus has been used to treat R/R marginal zone lymphomas (MZLs) with an ORR of 25% (75). Temsirolimus and everolimus are not very effective in lymphoma, which may be due to inhibition of mTORC1 but not mTORC2, resulting in feedback activation of PI3K/Akt (84). As for chronic lymphocytic leukemia (CLL), Decker et al. found that Rapamycin mediates the arrest of B cells of CLL in G1 phase (85). Three cases of lymphoproliferative diseases after transplantation have been successfully treated with rituximab and Rapamycin (86). Clinical evidence indicate that everolimus has a positive effect in patients with lymphoplasmacytic lymphoma/Waldenström macroglobulinemia (LPL/WM) and ORR can reach 70% (87). Taken together, subtypes of lymphoma may respond differently to mTORi; additional studies are needed to clarify the benefit/risk ratio of mTORi use in these settings.

### Multiple Myeloma

Multiple myeloma (MM) is a hematological malignancy characterized by abnormal production of immunoglobulins by malignant plasma cells (PCs). Previous studies have indicated that the PI3K/Akt/mTOR signaling pathway is aberrantly activated in MM cells (88, 89). MM blast cells secrete growth factor IL-6 and IGF-1 to activate PI3K/Akt, which induce abnormal expression of mTOR (90). Following curcumin treatment, mRNA and protein expression levels of mTOR were decreased, inducing apoptosis in MM cell lines, indicating a potential novel therapy for MM (91). Lamanuzzi A et al. (92) evaluated endothelial cells (ECs) from 20 patients with monoclonal gammopathy of undetermined significance (MGUS) and 47 patients with MM, and found higher activation of mTORC2 downstream effectors, suggesting a major role of mTORC2 in the angiogenic switch to MM. Inhibition of mTORC2 with lenalidomide and bortezomib exhibited a synergistic anti-angiogenic effect (92). Combining rapamycin with resveratrol has a synergistic effect in inhibition of myeloma cell line viability (93). Everolimus shows synergistic anti-myeloma effects with bortezomib through inhibition of the Akt/mTOR pathway in both the MM cell lines and MM-bearing mice model (94, 95).

In a clinical trial, 17 patients with R/R MM were administered single-agent everolimus; the ORR was 59% including eight patients with stable disease (SD), one with partial response (PR), one with minor remission (MR) (96). In another trial, 26 patients were administered everolimus and lenalidomide for 21 days with a 7 days break between cycles. The ORR was 65%, the median PFS was 5.5 months and median overall survival (OS) was 29.5 months (97). Temsirolimus has also been combined with lenalidomide in a phase I study in patients with relapsed MM. In total, 21 patients were enrolled, two patients (10%) achieved PR, and 15 patients (71%) had SD (98). In another phase I/II study evaluated temsirolimus in combination with weekly bortezomib in R/R MM patients, 14 of 43 (33%) patients achieved ORR (99). There are currently multiple approved drugs to treat multiple myeloma in front-line therapy; however, there is still no cure for this serious disease. Thus, more studies are needed to evaluate the potential benefit of incorporating mTORi into late-line regimens in R/R MM patients.

## Applications in Non-Malignant Hemopathies

### Immune Thrombocytopenic Purpura

The pathogenesis of immune thrombocytopenic purpura (ITP) includes abnormal activation of autoreactive T and B cells, which leads to immune destruction of platelets, providing a theoretical basis for the application of rapamycin in ITP. Wang CY et al. (100) found that platelet autophagy was diminished in ITP patients, and platelet autophagy in ITP was regulated by the PI3K/Akt/mTOR pathway. Rapamycin induced autophagy and alleviated the destruction of ITP platelets (100). Rapamycin and prednisone were used to treat adult chronic ITP (101). After comparing the remission rates of rapamycin group and cyclosporine group, as well as the levels of Treg cells, IL-10, and TGF - β cytokines in responders, it was concluded that rapamycin combined with low-dose dexamethasone could not improve the remission rate, but the sustained remission time of those treated with Rapamycin was significantly longer than that of cyclosporine-treated patients, and the Rapa-responders’ Treg cell level and Foxp3 mRNA expression level were significantly higher than those treated with cyclosporine.

Bride et al. (102) conducted a multicenter prospective study (NCT00392951) on rapamycin as a single therapy for refractory autoimmune pancytopenia of 30 patients. The result showed that rapamycin had a significant effect on secondary multicellular hemocytopenia, such as secondary Hypogammaglobulinemia, Evans Syndrome (ES), and SLE (systemic lupus erythematosus), especially thrombocytopenia, with a CR rate of 67% (8/12). In this report, all children (12) with autoimmune lymphoproliferative syndrome (ALPS) achieved a durable CR, including rapid improvement in autoimmune disease, lymphadenopathy, and splenomegaly within 1–3 months of starting sirolimus. Double-negative T cells were no longer detectable in most, suggesting a targeted effect of sirolimus. Jasinski et al. (103) treated 17 patients with sirolimus, among which were 12 ITP and 5 ES cases. As a result, 73% of ITP patients achieved a CR by 3 months, while 50% of ES patients had a CR. Of the patients that achieved CR, 90% remained off all therapy for a median of 2 years.

Miano et al. (104) retrospectively analyzed primary ITP (10 cases) and ITP secondary to autoimmune lymphoproliferative syndrome (nine cases) who received sirolimus treatment, and had previously failed mycophenolate mofetil (MMF) therapy. As a result, 5/10 primary ITP patients (50%) and 8/9 (89%) secondary ITP patients responded to SRL respectively. Our center initialed a prospective, single-arm clinical trial, in which 86 patients were included and given SRL administration. The results showed that SRL treatment obtained an ORR of 85% at the 3^rd^ month without serious drug toxicity. After 12 months follow-up, the ORR remained at 65%. Importantly, in patients who responded, SRL treatment was associated with a reduction in the percentage of Th2, Th17 cells, and increase in the percentage of monocytic-Myeloid-Derived Suppressor Cells (M-MDSCs) and T regulatory cells (Tregs), indicating that SRL may re-establish peripheral tolerance. To date, this is the largest prospective study to date evaluating SRL as a rescue therapy in patients with R/R ITP (105). ITP is a heterogeneous disease with a complex pathobiology; based on our experience and others’, we believe that mTORi can be effective in treating refractory ITP, but more randomized controlled trials (RCTs) are needed to support these observations.

### Autoimmune Hemolytic Anemia

Sirolimus has benefited patients with primary autoimmune cytopenias, possibly by stimulating Tregs (105, 106). In the pathogenesis of AIHA, increased expansion or function of Treg cells can inhibit the over-activation of effector T cells and other immune cells, thus regulating the production of excess RBC antibodies (107, 108). Mqadmi et al. (109) used CD25 monoclonal antibody to treat C57/B16 mice to deplete CD4^+^CD25^+^ Treg cells *in vivo*, followed by injection allogeneic red blood cells into the abdominal cavity of C57/B16 mice to establish a murine AIHA model. These experiments showed that incidence of AIHA in Treg deficient mice increased from 30% to 90%. CD4^+^CD25^+^ Treg cells purified from the spleen of mice were re-injected into the diseased mice, leading to a reduction in hemolysis and autoantibodies. Thus, CD4^+^ CD25^+^ Treg cells play an important role in the pathogenesis of AIHA in animal models (110, 111).

Miano et al. reported that sirolimus was effective in five children with AIHA who did not respond to MMF previously. In their center, sirolimus is used as a rescue treatment, given at the dose of 2–3 mg/m^2^ once a day, for at least 3 month (112). In refractory AIHA after intestinal transplant, four patients were reported who had marked improvement of hemolysis, after discontinuation of calcineurin inhibitor (CNI) and initiation of sirolimus (113). It was recommended sirolimus as the second-line treatment of AIHA in the case of failure to steroid treatment in 2016 of ASH (American Society of Hematology) meeting (114). Our center used sirolimus to treat the relapsed and refractory AIHA. Sixteen (16) patients were included and followed for at least 12 months; after 12 months, SRL was still effective for 9/12 patients (56.25%), with 7/9 patients achieving CR and 2/9 achieving PR. Five (5) of these patients had been discontinued with no relapse, and the longest withdrawal time without relapse was 24 months. The side effects of SRL were mild and tolerable to the patients. In conclusion, SRL is effective for R/R AIHA in adults, with mild side effects, and should be considered as frontline therapy for treatment of the disease (115).

### Autoimmune Lymphoproliferative Syndrome

Autoimmune lymphoproliferative syndrome (ALPS) is mainly caused by mutations in FAS mediated apoptosis pathway (116, 117). The pathogenesis of ALPS is that the double negative T cells (DNT), expressing CD3^+^TCRαβ^+^ CD4^-^CD8^-^, cannot be effectively removed due to apoptosis defects caused by FAS mutation, resulting in abnormal activation of immature T cell accumulation causes hepatosplenic lymphadenopathy. At the same time, over-activation of B lymphocyte leads to the increase of autoantibody production and the occurrence of autoimmunity (118). mTOR signaling activity was also enhanced in DNT cells, and mTOR inhibitors could specifically reduce DNT *in vivo*, indicating that the mTOR pathway is the main regulatory mechanism of lymphocyte proliferation and abnormal differentiation of ALPS (119). In 2006, the results of an experimental study in animals provided the first evidence that SRL can significantly improve the clinical symptoms of ALPS rats (120). Sirolimus has been described as useful in ALPS patients, in which it also reduced the count of DNTs (121).

Currently, there are some cases of successful application of sirolimus to control ALPS in adults and children (102, 122, 123). In one case-study, a 9-month-old female infant diagnosed ALPS with massive lymphadenopathy was treated with corticosteroids and splenectomy, but the curative effect was poor. Treatment with SRL (3 mg/m^2^) was initiated and the symptoms of lymphadenopathy were relieved rapidly. After 6 weeks, lymphadenopathy decreased and blood cell count improved significantly (124). In another center, four steroid-resistant ALPS patients were treated with SRL for 4 weeks; all had a rapid complete or near complete response (123). SRL was used as single agent in 12 ALPS patients for at least 6 months. The results showed that SRL was effective in all ALS patients. It not only improved the decreased blood cells, but also improved the hyperplasia of lymphoid tissue (102). In a report, 16 ALPS patients were treated with SRL as the second or further line, after the multi-drug treatment failed. As a result, 12 (75%) patients responded effectively (125). Recently, IL-6 stimulation augmented mTOR activation in idiopathic multicentric Castleman disease (iMCD) patients was reported, furthermore, the degree of mTOR activation in iMCD was comparable to ALPS. This finding supports inhibition of mTOR activation as a novel therapeutic target for iMCD, and is currently under investigation in a clinical study (NCT03933904) (126).

### Acquired Aplastic Anemia and Pure Red Cell Aplasia

Acquired aplastic anemia is characterized by a hypoplastic, fatty bone marrow with profound reductions in hematopoietic stem/progenitor cells that lead to defective mature blood cell production and peripheral pancytopenia (127). In clinical observation, it was found that the IFN-γ level in serum and bone marrow of was increased in ~30% of AA patients increased, while cytotoxic T cells (CD8^+^ T cells) production of IFN-γ promoted CD34^+^ pluripotent hematopoietic progenitor cell apoptosis (128). The attack of CD34^+^ cells by CD8^+^ T cells is the basis of AA, the effect of CD8^+^ T cells can be eliminated by SRL mediated CD4^+^CD25^+^ Treg cells, which inhibit the process and promote the proliferation of hematopoietic stem cells (129, 130). Rapamycin ameliorated this phenotype in an immune-mediated AA mice model and inhibited the proliferation of T cells by preventing cell cycle transition from G0 to G1 phase (131). Reports also show that rapamycin can significantly enhance autophagy of Bone Marrow Stromal Cells (BMSCs). Rapamycin can inhibit the adipogenic differentiation of BM-MSCs in AA patients in a dose-dependent manner, and the inhibition rate can reach 50%-85%. Rapamycin can reduce the ratio of adipose tissue to bone marrow hematopoietic tissue, so as to improve the hematopoietic microenvironment of bone marrow. However, SRL may also promote the apoptosis of MSC; accordingly, rapamycin should be strictly controlled in clinical treatment (132). He et al. reported two case of AA responsive to sirolimus (1 mg/d) combined with cyclosporine (2 mg/kg/d) (133). In fact, controversial results are also presented. A study observed 35 AA patients received h-ATG/CsA/sirolimus, and the ORR at 3 months was 37% and 51% at 6 months, at last, it concluded that sirolimus (2 mg/day in adults and 1 mg/m^2^/day in children) did not improve the response rate in patients with severe AA when compared to standard h-ATG/CsA treatment (134).

Sirolimus may not directly stimulate the growth and differentiation of red blood cells, but it can antagonize the inhibitory effect of PRCA patients’ serum on red blood cells, and may play a role by inhibiting the components in serum (135). In 2007, ASH meeting, Sirolimus (1 mg/day) successfully treated a refractory case of congenital PRCA was reported (136). Reportedly, three patients of multi-resistant PRCA treated with SRL (2 mg/day), all responded well to SRL, and natural killer cell (CD16^+^CD56^+^) count was decreased after long-term administration of sirolimus (137). In 2018, 21 patients with refractory/relapsed acquired PRCA administered with SRL, and 76.2% of the patients was responsive and 42.9% was complete response (138). At present, there is still a need for multi-center and large-scale RCT to verify the positive role of rapamycin in acquired AA and PRCA.

### Graft-Versus-Host Disease

SRL has been widely used to inhibit graft rejection after solid organ transplantation; however, increasing evidence demonstrate that SRL plays an active role in the prevention and treatment of graft-versus-host disease (GVHD) following hematopoietic stem cell transplantation (HSCT). GVHD is an exaggerated inflammatory reaction mediated by donor lymphocytes against host tissues. The conditioning regimen damages host tissues and causes release of pro-inflammatory soluble factors such as TNFα, IFN-γ, IL-1, IL-6, and nitric oxide. Increased levels of these factors lead to activation of host antigen presenting cells (APCs). Host APCs migrate into lymphoid tissue and subsequently activate donor immune cells, including T, B, and NK cells. This process elicits a complex cascade of both lymphocyte subsets and soluble inflammatory mediators. Lastly, these soluble and cellular mediators synergize to amplify local tissue injury and further promote inflammation and target tissue destruction (139, 140). Coenen JJ et al. (141), reported rapamycin, not cyclosporine, permits thymic generation and peripheral preservation of CD4^+^CD25^+^FoxP3^+^ T cells. Treatment with cyclosporine led to a reduced generation of CD4^+^FoxP3^+^ T cells in GVHD mice model, whereas prolonged rapamycin treatment allowed for thymic generation of those cells. As peripheral tolerance induction is a prerequisite for successful treatment outcome after stem cell transplantation (SCT), this may challenge the use of cyclosporine as standard drug of choice (141).

In 2008, Armand et al. (142) found that sirolimus combined with calcineurin inhibitor prevented GVHD in lymphoma patients after bone marrow transplantation. The OS of patients in sirolimus group was significantly prolonged (3-year OS, 66% for sirolimus group vs. 38% for no sirolimus group). In 2016, Armand and his colleagues (143), again reported a multicenter randomized trial comparing sirolimus, tacrolimus and methotrexate to standard method (tacrolimus, methotrexate and mycophenolate mofetil) in lymphoma patients after SCT. As a result, there was no difference in 2-year OS, PFS, relapse, and chronic GVHD. However, the sirolimus-containing arm had a significantly lower incidence of grade II‐IV acute GVHD. In 2019, another multi-center, randomized, phase 3 trial was reported. After HSCT, standard GVHD prophylaxis regimen (cyclosporine and mycophenolate mofetil) or the triple-drug combination regimen (cyclosporine, mycophenolate mofetil, and sirolimus) was used to test the efficacy of SRL. Consistent with previous findings, the incidence of II‐IV aGVHD in the three-drug group (26%) was lower than that in the standard group (52%) at 100 days. The new discoveries were as following: The Non Recurrent Mortality (NRM) rate (16%) of the three drug groups was lower than that of the standard group (32%) (*p*=0.021) at 4 years; The OS rate in the three drug groups was higher than that in the standard group (*p*=0.035); The PFS rates in the three drug groups were higher than those in the standard group (*p*=0.045); The incidence of Grade III to IV aGVHD and chronic GVHD was of no differences (144). A meta-analysis included five RCTs to assess the efficacy and safety of sirolimus-based GVHD prophylaxis in patients after allogeneic HSCT, as the result, SIR was observed to significantly decrease the incidence of Grades II to IV aGVHD; However, the incidence of Grades III to IV aGVHD and chronic GVHD was not decreased. SRL significantly increased sinusoidal obstructive syndrome, Moreover, SRL did not improve event-free survival and overall survival (145).

In addition to the above reports on the prevention of GVHD by SRL, the following is the story of using sirolimus to treat GVHD. Pidala et al. (146) initiated a multicenter randomized phase II trial to assess the CR/PR rates of SRL versus prednisone to treat patients with acute GVHD of standard risk. A total of 127 patients were enrolled and randomized. The day 28 CR/PR rates were similar for sirolimus 64.8% vs. 73% for prednisone. Interestingly, the day 28 rate of CR/PR with prednisone (≤0.25 mg/kg/day) was significantly lower than sirolimus (31.7% vs. 66.7%; *P*<0.001). Importantly, SRL was associated with reduced steroid exposure and hyperglycemia, reduced grades 2 to 3 infections, improvement in immune suppression discontinuation and patient-reported quality of life. For chronic GVHD treatment, Carpenter PA et al. (147), claimed that the two-drug (prednisone/sirolimus) and three-drug combination (prednisone/sirolimus/CNI) did not differ in rates of 6-month CR and PR. In other words, the results are the same whether CNI is not added to the two-drug regimen. Importantly, the two-drug regimen is easier to administer and is better tolerated. In our center, sirolimus was used to treat steroid- resistant/steroid- dependent extensive cGVHD. A total of 27 patients were enrolled and given sirolimus combined with cyclosporine or tacrolimus to observe the clinical efficacy and adverse events. Following the 6-month follow-up, the ORR was 55.6%. At the 1-year follow-up, there were five cases of CR and 11 cases of PR, ORR was 59.3%, PFS-12 reached 62.9% (17/27), and OS-12 was 100% (148).

In summary, SRL has demonstrated clinical benefit in both the prevention and treatment of GVHD and may be preferred to other regimens for patients after HSCT. There is currently extensive clinical investigation of Rapalogs in the GVHD setting (**Table 1**).

**Table 1:** Current recruiting Rapalog clinical trial landscape in hematologic diseases.

	Registration Number	Rapalogs	Phase	Disease	Status	Interventions
**1**	NCT03963024	Sirolimus	1	GVHD	Recruiting	Sirolimus
**2**	NCT03225417	Sirolimus	1/2	GVHD	Recruiting	Sirolimus + Ixazomib + Tacrolimus
**3**	NCT02891603	Sirolimus	1/2	GVHD	Recruiting	Sirolimus + Pacritinib + Tacrolimus
**4**	NCT03128034	Sirolimus	1/2	GVHD	Recruiting	Sirolimus + CsA + MMF
**5**	NCT03192397	Sirolimus	2	GVHD	Recruiting	Sirolimus + MMF +PTCY
**6**	NCT03970096	Sirolimus	2	GVHD	Recruiting	Sirolimus + Tacrolimus + MTX+ATG
**7**	NCT01903473	Sirolimus	2	GVHD	Recruiting	Sirolimus + Treg infusion + Low dose IL-2
**8**	NCT02722668	Sirolimus	2	GVHD	Recruiting	Sirolimus + MMF + ATG
**9**	NCT03246906	Sirolimus	2	GVHD	Recruiting	Sirolimus + CsA + MMF vs. Sirolimus + CsA + PTCY
**10**	ChiCTR2000029921	Sirolimus	2	GVHD	Recruiting	Sirolimus + CNI
**11**	NCT02583893	Sirolimus	2	AML	Recruiting	Sirolimus + Mitoxantrone + Etoposide + Cytarabine
**12**	NCT03878524	Sirolimus, Everolimus	1/2	MDS, PMF	Recruiting	Sirolimus for MDS; Everolimus for PMF
**13**	NCT03697408	Everolimus	1/2	cHL	Recruiting	Everolimus + Itacitinib
**14**	NCT03190174	Nab-rapamycin	1/2	cHL	Recruiting	Nab-rapamycin + Nivolumab
**15**	NCT02693535	Temsirolimus	2	NHL, MM	Recruiting	Temsirolimus
**16**	ChiCTR1900020657	Sirolimus	2	ITP	Recruiting	Sirolimus + Dexamethasone

PTCY, Post-Transplantation Cyclophosphamide; MMF, mycophenolate mofetil; MTX, methotrexate; ATG, Antithymocyte globulin; CNI, Calcineurin Inhibitor; CsA, ciclosporin; PMF, Primary myelofibrosis; cHL, classical Hodgkin lymphoma; Nab-rapamycin, Nanoparticle albumin-bound rapamycin.

The data are from https://www.clinicaltrials.gov/in America and www.chictr.org.cn in China. This table only includes the recruiting trails, and the “active not recruiting” and “completed” are excluded.

## Conclusions and Future Directions

Rapalogs, as inhibitors of mTOR, have demonstrated clinical value, not only in the regulation of abnormal immune responses found in benign hematologic diseases, but also for the treatment of hematological malignancies (**Table 2**). However, there are still some challenges in the application of Rapalogs. First, at present, most of the studies of Rapalogs are case reports of three-line or further line drugs, which are not widely used in large-scale populations, so there remains a lack of evidence—effectiveness and long-term safety of Rapalogs need to be further studied. Whether SRL can be used as a more advanced drug in the treatment paradigm(s) needs to be evaluated by more extensive clinical research. Second, it is necessary to improve the efficacy and safety of Rapalogs, establishing the proper dosing regiments, maintaining appropriate blood concentrations model to optimize the current treatment. Third, many cytotoxic drugs are limited in the pediatric setting. The question arises, can Rapalogs demonstrate an advantage over current therapies in convenience, safety, and efficacy on pediatric patients? Finally, in clinical studies to date, we found that there are inevitably some patients that do not responded to Rapalogs, irrespective of primary disease. Can we achieve precise treatment? Before treatment, can we accurately stratify patients which are likely responders to Rapalogs? Perhaps by detecting the activity of mTOR, specific mutations responsible of driving mTOR activity or a deeper prognostic biomarker? Additional work is required in this exciting and evolving area.

**Table 2:** Clinical efficacy and safety of mTOR inhibitors in hematological disease.

Author (Ref)	Disease(s)	Patients (n)	Drugs and Schedules	Clinical Response	Adverse events (AEs)
Kasner MT. 2018 (36)	Relapsed, refractory, or untreated high-risk AML	51	Sirolimus 12 mg d1, 4 mg d2-9; Mitoxantrone 8 mg/m^2^, 5days; Etoposide 100 mg/m^2^, 5 days; Cytarabine 1 g/m^2^, 5 days.	ORR was 47% (33% CR, 2% CRi, 12% PR), Blood p70S6 as a predictive biomarker for clinical response.	No significant AEs.
Perl AE. 2009 (37)	Relapsed, refractory, or untreated secondary AML	27	Sirolimus 12 mg d1, 4 mg d2-9; Mitoxantrone 8 mg/m^2^, 5 days; Etoposide 100 mg/m^2^, 5 days; Cytarabine 1 g/m^2^, 5 days.	ORR was 22% (15% CR, 7% PR), p70S6 as a biomarker for SRL response.	Neutropenic fever, Bacteremia, Diarrhea, Transaminase/bilirubin, elevation, Pneumonia
Daver N. 2015 (32)	Relapsed/Refractory ALL	24	HyperCVAD combined with continuous oral everolimus, starting on day 0 of Cycle 1, at a dose of either 5 or 10 mg/day.	ORR was 41% (33% CR, 8% PR and NR 59%), Everolimus inhibited phosphorylation of S6RP, but did not correlate with response.	Mucositis, Myelosuppression, Hyperglycemia, Transaminitis
Guglielmelli P. 2011 (55)	MPN	39	Everolimus in 3 dose-escalating cohorts at 5.0, 7.5, and 10.0 mg daily for 3 months.	Response rate was 60%, whereas CCDN1 mRNA, phospho-p70S6K level, and WT1 mRNA were identified as possible biomarkers.	Stomatitis, Hypertriglyceridemia, Skin disorders, Musculoskeletal, Hypercholesterolemia
Johnston PB. 2012 (68)	Hodgkin lymphoma	57	Everolimus 10 mg/day until disease progression or unacceptable toxicity.	The ORR and DCR were 42.1% and 77.2%, including CR 8.8%. The median time to response was 57 days, Median PFS was 9.0 months.	Fatigue, Thrombocytopenia, Cough, Rash, Pyrexia, Anemia, Dyspnea, Back pain, Diarrhea, Stomatitis, Pneumonitis.
Janku F. 2014 (77)	Refractory Hodgkin lymphoma	28	Sirolimus 1–5 mg/day q28 days; HDAC inhibitor vorinostat 100–400 mg/day q28 days.	The ORR was 57% with 9 CRs (32%) and 7 PRs (25%).	Thrombocytopenia, Anemia, Transaminitis.
Tessoulin B. 2020 (81)	Relapsed MCL	41	15, 25, and 50 mg dose level plus RCHOP, or RDHA, or RFC	The ORR was 40% in RCHOP-T arm, 43% RFC-T arm, and 47% in RDHA-T arm.	Blood count disorders, metabolism troubles, biologic investigations, and infections.
Smith SM 2010 (83)	Relapsed DLBCL, FL and CLL	89	Single-agent temsirolimus 25 mg weekly	Group A (DLBCL) had an ORR of 28.1% and median PFS of 2.6 months. Group B (FL) had ORR of 53.8% median PFS of 12.7 months; Group C (CLL) had ORR of 11%.	Mild and/or reversible myelosuppression and mucositis
Ghobrial IM. 2010 (87)	WM	50	Everolimus 10 mg/day until progression.	The ORR was 70%, with a PR of 42% and 28% MR. CR 0.	Anemia, Leukopenia, Neutropenia, Pneumonia, Hypercholesterolemia, Hyperglycemia.
Yee AJ. 2014 (97)	Relapsed/refractory multiple myeloma	26	Lenalidomide 15 mg; Everolimus 5 mg for 21 days with a 7-day rest period.	The ORR was 65% (1 CR + 4 PR + 10 MR). The median PFS was 5.5 months and median OS was 29.5 months. Phosphorylated p70S6K at Thr389 as biomarker.	Fatigue, Neutropenia, Thrombocytopenia, Neuropathy, and diarrhea, all of which were manageable with supportive care and dose‐reductions.
Hofmeister CC. 2011 (98)	Relapsed/refractory multiple myeloma	21	Lenalidomide 15–25 mg/d d1-d21; Temsirolimus 15–20 mg once per week during a 28-day cycle.	Two patients (10%) achieved PR and 15 patients (71%) had SD.	Fatigue, Neutropenia, Anemia, Hypophosphatemia, Hypokalemia, Rash, Hypokalemia, Hypophosphatemia, Anorexia, Nausea, Taste alterations.
Ghobrial IM. 2011 (99)	Relapsed/refractory multiple myeloma	20	Temsirolimus 15 or 25 mg; Bortezomib at 1.3 or 1.6 mg/m^2^ once a week, with dose escalation until dose-limiting adverse events were recorded.	14 of 43 (33%) patients achieved ORR.	Thrombocytopenia, Lymphopenia, Neutropenia Leucopenia, Anemia, Diarrhea.
Feng YM. 2020 (105)	Relapsed/refractory ITP	86	Sirolimus 2–4 mg/day.	By the third month, 40% CR and 45% PR, whereby (ORR) of 85%. Percentage of Th2, Th17 cells, M-MDSCs, and Tregs as biomarker associated with response.	Hyperlipidemia, Hypertransaminase, Canker sores, Skin rash, Arthralgia.
Miano M. 2015 (125)	ALPS, ALPS-related syndrome, and autoimmune cytopenia.	16	Sirolimus 2–3 mg/m^2^/day, at least 3M	12/16 children ORR75%, 11 with CR (69%), 1 with PR (6%).	Patients did not show an increased incidence of opportunistic infections with mild headache being the most common side effect.
Long ZB. 2018 (138).	Refractory/relapsed acquired PRCA	21	Sirolimus 1–3 mg/day, at least 6M.	The ORR was 76.2%, with a CR of 42.9%. Tregs as biomarker associated with response.	Pneumonia, elevation of creatinine, mild elevation of transaminase, oral mucositis, sinus tachycardia, and elevation of triglyceride and cholesterol.
Wang L. 2015 (145)	Acute GVHD and chronic GVHD	395	(TAC, SRL and MTX or MMF) TAC: -3 days, 0.02 mg/kg/day, 5–10 ng/ml 100 days tapered; SRL: 2 mg qd (-3 days _˜_ +80 days), 3–12 ng/ml; MTX: +1 day (15 mg/m^2^),+3 days,+6 days, +11 days (10 mg/m^2^); or MMF: +0 day _˜_ +30 days (15 mg/kg tid), 30–40 days (15 mg/kg bid), in the absence of GVHD, tapered off by day 96 to day 150.	SRL significantly decrease the incidence of Grades II to IV aGVHD, but has no effect in decreasing cGVHD. SRL did not improve EFS and OS.	Sirolimus increased in the incidence of SOS and TMA.
Pidala J, 2020 (146).	Acute GVHD	58	Prednisone 2 mg/kg/day; SRL was given as a loading dose (6 mg for those aged >12 years, 5 mg/m^2^ for those aged ≤ 12 years), at least 56 days (10–14 ng/ml until acute GVHD resolution, then 5–10 ng/ml after resolution until at least day 56).	Day 28 CR/PR rates were similar for sirolimus 64.8% vs. 73% for prednisone. The day 28 rate of CR/PR with prednisone ≤0.25 mg/kg/day was significantly higher for sirolimus than prednisone (66.7% vs. 31.7%). No differences were detected in DFS, relapse, NRM, and OS.	Hyperglycemia was lower in the sirolimus group. The rate of TMA within 6 months was higher in the sirolimus group vs the prednisone group.

SRL, Sirolimus; CR, Complete remission; CRi, complete remission with incomplete hematologic recovery; PR, Partial remission; ORR, Overall response rate; DCR, Disease control rate; MR, Minimal response; SD, Stable disease; HDAC, Histone deacetylase; MDSC, Myeloid derived suppressor cells; SOS, Sinusoidal obstruction syndrome; TMA, Thrombotic Microangiopathy; TAC, tacrolimus; MTX, methotrexate; MMF, Mycophenolate mofetil; DFS, Disease-free Survival; NRM, Non-relapse Mortality; OS, Overall Survival.

## Author Contributions

YF wrote the original draft and revised the manuscript. XC, KC, ZZ, ZW, SY, and XZ edited this review. XZ and YF: funded the work. All authors contributed to the article and approved the submitted version.

## Funding

This work was supported by the Science and Technology Innovation Capacity Promotion Project of Army Medical University (2019XLC3014), Natural Science Foundation of Chongqing (cstc2019jcyj-msxmX0273 and cstc2020jcyj-msxmX1086), Special Projects in the Frontier of Military Medicine Natural Science of Xinqiao Hospital (2018YQYLY002), National Key Research Program (2017YFA0105502).

## Conflict of Interest

The authors declare that the research was conducted in the absence of any commercial or financial relationships that could be construed as a potential conflict of interest.

## References

[1] Kelchtermans J Chang J Glaberson W DeFreitas M Alba-Sandoval M Chandar J A Pediatric Case of Sirolimus-Associated Pneumonitis After Kidney Transplantation. J Pediatr Pharmacol Ther (2020) 25:459–64. 10.5863/1551-6776-25.5.459 PMC7337132 32641918

[2] Dufour M Dormond-Meuwly A Demartines N Dormond O Targeting the Mammalian Target of Rapamycin (mTOR) in Cancer Therapy: Lessons from Past and Future Perspectives. Cancers (Basel) (2011) 3:2478–500. 10.3390/cancers3022478 PMC3757428 24212820

[3] Tan FH Bai Y Saintigny P Darido C mTOR Signalling in Head and Neck Cancer: Heads Up. Cells (2019) 8:333. 10.3390/cells8040333 PMC6523933 30970654

[4] Adjei AA Hidalgo M Intracellular signal transduction pathway proteins as targets for cancer therapy. J Clin Oncol (2005) 23:5386–403. 10.1200/JCO.2005.23.648 15983388

[5] Sarbassov DD Ali SM Kim DH Guertin DA Latek RR Erdjument-Bromage H Rictor, a novel binding partner of mTOR, defines a rapamycin-insensitive and raptor-independent pathway that regulates the cytoskeleton. Curr Biol (2004) 14:1296–302. 10.1016/j.cub.2004.06.054 15268862

[6] Laplante M Sabatini DM mTOR signaling in growth control and disease. Cell (2012) 149:274–93. 10.1016/j.cell.2012.03.017 PMC3331679 22500797

[7] Pearce LR Sommer EM Sakamoto K Wullschleger S Alessi DR Protor-1 is required for efficient mTORC2-mediated activation of SGK1 in the kidney. Biochem J (2011) 436:169–79. 10.1042/BJ20102103 21413931

[8] Sarbassov DD Ali SM Sengupta S Sheen JH Hsu PP Bagley AF Prolonged rapamycin treatment inhibits mTORC2 assembly and Akt/PKB. Mol Cell (2006) 22:159–68. 10.1016/j.molcel.2006.03.029 16603397

[9] Meng LH Zheng XF Toward rapamycin analog (rapalog)-based precision cancer therapy. Acta Pharmacol Sin (2015) 36:1163–9. 10.1038/aps.2015.68 PMC4648176 26299952

[10] Malagelada C Jin ZH Jackson-Lewis V Przedborski S Greene LA Rapamycin protects against neuron death in in vitro and in vivo models of Parkinson’s disease. J Neurosci (2010) 30:1166–75. 10.1523/JNEUROSCI.3944-09.2010 PMC2880868 20089925

[11] Ma KL Ruan XZ Powis SH Moorhead JF Varghese Z Anti-atherosclerotic effects of sirolimus on human vascular smooth muscle cells. Am J Physiol Heart Circ Physiol (2007) 292:H2721–8. 10.1152/ajpheart.01174.2006 17322416

[12] Goudar RK Shi Q Hjelmeland MD Keir ST McLendon RE Wikstrand CJ Combination therapy of inhibitors of epidermal growth factor receptor/vascular endothelial growth factor receptor 2 (AEE788) and the mammalian target of rapamycin (RAD001) offers improved glioblastoma tumor growth inhibition. Mol Cancer Ther (2005) 4:101–12. 15657358

[13] Liu Y Zhang DT Liu XG mTOR Signaling in T Cell Immunity and Autoimmunity. Int Rev Immunol (2015) 34:50–66. 10.3109/08830185.2014.933957 25019278

[14] Cutler C Antin JH Sirolimus immunosuppression for graft-versus-host disease prophylaxis and therapy: an update. Curr Opin Hematol (2010) 17:500–4. 10.1097/MOH.0b013e32833e5b2e 20717025

[15] Chapuis N Tamburini J Green AS Willems L Bardet V Park S Perspectives on inhibiting mTOR as a future treatment strategy for hematological malignancies. Leukemia (2010) 24:1686–99. 10.1038/leu.2010.170 20703258

[16] Kirchner GI Meier-Wiedenbach I Manns MP Clinical pharmacokinetics of everolimus. Clin Pharmacokinet (2004) 43:83–95. 10.2165/00003088-200443020-00002 14748618

[17] Kovarik JM Everolimus - A proliferation signal inhibitor targeting primary causes of allograft dysfunction. Drugs Today (2004) 40:101–9. 10.1358/dot.2004.40.2.799422 15045032

[18] Sessa C Tosi D Vigano L Albanell J Hess D Maur M Phase Ib study of weekly mammalian target of rapamycin inhibitor ridaforolimus (AP23573; MK-8669) with weekly paclitaxel. Ann Oncol (2010) 21:1315–22. 10.1093/annonc/mdp504 19901013

[19] Chawla SP Staddon AP Baker LH Schuetze SM Tolcher AW D’Amato GZ Phase II study of the mammalian target of rapamycin inhibitor ridaforolimus in patients with advanced bone and soft tissue sarcomas. J Clin Oncol (2012) 30:78–84. 10.1200/JCO.2011.35.6329 22067397

[20] Seiler M Ray-Coquard I Melichar B Yardley DA Wang RX Dodion PF Oral ridaforolimus plus trastuzumab for patients with HER2+ trastuzumab-refractory metastatic breast cancer. Clin Breast Cancer (2015) 15:60–5. 10.1016/j.clbc.2014.07.008 25239224

[21] Rizzieri DA Feldman E Dipersio JF Gabrail N Stock W Strair R A phase 2 clinical trial of deforolimus (AP23573, MK-8669), a novel mammalian target of rapamycin inhibitor, in patients with relapsed or refractory hematologic malignancies. Clin Cancer Res (2008) 14:2756–62. 10.1158/1078-0432.CCR-07-1372 18451242

[22] Serruys PW Silber S Garg S van Geuns RJ Richardt G Buszman PE Comparison of zotarolimus-eluting and everolimus-eluting coronary stents. N Engl J Med (2010) 363:136–46. 10.1056/NEJMoa1004130 20554978

[23] Chan S Scheulen ME Johnston S Mross K Cardoso F Dittrich C Phase II study of temsirolimus (CCI-779), a novel inhibitor of mTOR, in heavily pretreated patients with locally advanced or metastatic breast cancer. J Clin Oncol (2005) 23:5314–22. 10.1200/JCO.2005.66.130 15955899

[24] Witzens-Harig M Memmer ML Dreyling M Hess G A phase I/II trial to evaluate the safety, feasibility and activity of salvage therapy consisting of the mTOR inhibitor Temsirolimus added to standard therapy of Rituximab and DHAP for the treatment of patients with relapsed or refractory diffuse large cell B-Cell lymphoma - the STORM trial. BMC Cancer (2013) 13:308. 10.1186/1471-2407-13-308 23799873 PMC3701613

[25] Pandya KJ Dahlberg S Hidalgo M Cohen RB Lee MW Schiller JH A randomized, phase II trial of two dose levels of temsirolimus (CCI-779) in patients with extensive-stage small-cell lung cancer who have responding or stable disease after induction chemotherapy: a trial of the Eastern Cooperative Oncology Group (E1500). J Thorac Oncol (2007) 2:1036–41. 10.1097/JTO.0b013e318155a439 17975496

[26] Dancey JE Curiel R Purvis J Evaluating temsirolimus activity in multiple tumors: a review of clinical trials. Semin Oncol (2009) 36 Suppl 3:S46–58. 10.1053/j.seminoncol.2009.10.010 19963100

[27] Feng YH Wu LS mTOR up-regulation of PFKFB3 is essential for acute myeloid leukemia cell survival. Biochem Biophys Res Commun (2017) 483:897–903. 10.1016/j.bbrc.2017.01.031 28082200

[28] Xu Q Simpson SE Scialla TJ Bagg A Carroll M Survival of acute myeloid leukemia cells requires PI3 kinase activation. Blood (2003) 102:972–80. 10.1182/blood-2002-11-3429 12702506

[29] Xu Q Thompson JE Carroll M mTOR regulates cell survival after etoposide treatment in primary AML cells. Blood (2005) 106:4261–8. 10.1182/blood-2004-11-4468 PMC1895255 16150937

[30] Brown VI Fang JJ Alcorn K Barr R Kim JM Wasserman R Rapamycin is active against B-precursor leukemia in vitro and in vivo, an effect that is modulated by Il-7-mediated signalling. Proc Natl Acad Sci U S A (2003) 100:15113–8. 10.1073/pnas.2436348100 PMC299917 14657335

[31] Avellino R Romano S Parasole R Bisogni R Lamberti A Poggi V Rapamycin stimulates apoptosis of childhood acute lymphoblastic leukemia cells. Blood (2005) 106:1400–6. 10.1182/blood-2005-03-0929 15878982

[32] Daver N Boumber Y Kantarjian H Ravandi F Cortes J Rytting ME A Phase I/II Study of the mTOR Inhibitor Everolimus in Combination with HyperCVAD Chemotherapy in Patients with Relapsed/Refractory Acute Lymphoblastic Leukemia. Clin Cancer Res (2015) 21:2704–14. 10.1158/1078-0432.CCR-14-2888 PMC4470787 25724525

[33] Recher C Beyne-Rauzy O Demur C Chicanne G Dos Santos C Mansat-De Mas V Antileukemic activity of rapamycin in acute myeloid leukemia. Blood (2005) 105:2527–34. 10.1182/blood-2004-06-2494 15550488

[34] Yee KWL Zeng ZH Konopleva M Verstovsek S Ravandi F Ferrajoli A Phase I/II study of the mammalian target of rapamycin inhibitor everolimus (RAD001) in patients with relapsed or refractory hematologic malignancies. Clin Cancer Res (2006) 12:5165–73. 10.1158/1078-0432.CCR-06-0764 16951235

[35] Litzow MR Wang XV Carroll MP Karp JE Ketterling RP Zhang YM A randomized trial of three novel regimens for recurrent acute myeloid leukemia demonstrates the continuing challenge of treating this difficult disease. Am J Hematol (2019) 94:111–7. 10.1002/ajh.25333 PMC6298814 30370956

[36] Kasner MT Mick R Jeschke GR Carabasi M Filicko-O’Hara J Flomenberg N Sirolimus enhances remission induction in patients with high risk acute myeloid leukemia and mTORC1 target inhibition. Invest New Drugs (2018) 36:657–66. 10.1007/s10637-018-0585-x PMC6060002 29607465

[37] Perl AE Kasner MT Tsai DE Vogl DT Loren AW Schuster SJ A Phase I Study of the Mammalian Target of Rapamycin Inhibitor Sirolimus and MEC Chemotherapy in Relapsed and Refractory Acute Myelogenous Leukemia. Clin Cancer Res (2009) 15:6732–9. 10.1158/1078-0432.CCR-09-0842 19843663

[38] Burnett AK Das Gupta E Knapper S Khwaja A Sweeney M Kjeldsen L Addition of the mammalian target of rapamycin inhibitor, everolimus, to consolidation therapy in acute myeloid leukemia: experience from the UK NCRI AML17 trial. Haematologica (2018) 103:1654–61. 10.3324/haematol.2018.189514 PMC6165825 29976746

[39] Robin M Fenaux P Which lower risk myelodysplastic syndromes should be treated with allogeneic hematopoietic stem cell transplantation? Leukemia (2020) 34(10):2552–60. 10.1038/s41375-020-0967-x 32661295

[40] Yip BH Vuppusetty C Attwood M Giagounidis A Germing U Lamikanra AA Activation of the mTOR signaling pathway by L-leucine in 5q-syndrome and other RPS14-deficient erythroblasts. Leukemia (2013) 27:1760–3. 10.1038/leu.2013.20 23337929

[41] Follo MY Mongiorgi S Bosi C Cappenini A Finelli C Chiarini F The Akt/mammalian target of rapamycin signal transduction pathway is activated in high-risk myelodysplastic syndromes and influences cell survival and proliferation. Cancer Res (2007) 67:4287–94. 10.1158/0008-5472.CAN-06-4409 17483341

[42] Maeda Y Yamaguchi T Ueda S Matsuo K Morita Y Naiki Y Mutant type glutathione S-transferase theta 1 gene homologue to mTOR in myelodysplastic syndrome: Possible clinical application of rapamycin. Leuk Lymphoma (2003) 44:1179–85. 10.1080/1042819031000077052 12916871

[43] Platzbecker U Haase M Herbst R Hanel A Voigtmann K Thiede CH Activity of sirolimus in patients with myelodysplastic syndrome–results of a pilot study. Br J Haematol (2005) 128:625–30. 10.1111/j.1365-2141.2005.05360.x 15725083

[44] Wermke M Schuster C Nolte F Al-Ali HK Kiewe P Schonefeldt C Mammalian-target of rapamycin inhibition with temsirolimus in myelodysplastic syndromes (MDS) patients is associated with considerable toxicity: results of the temsirolimus pilot trial by the German MDS Study Group (D-MDS). Br J Haematol (2016) 175:917–24. 10.1111/bjh.14345 27714772

[45] Annunziata M Bonifacio M Breccia M Castagnetti F Gozzini A Iurlo A Current Strategies and Future Directions to Achieve Deep Molecular Response and Treatment-Free Remission in Chronic Myeloid Leukemia. Front Oncol (2020) 10:883. 10.3389/fonc.2020.00883 32582549 PMC7280484

[46] Mayerhofer M Valent P Sperr WR Griffin JD Sillaber C BCR/ABL induces expression of vascular endothelial growth factor and its transcriptional activator, hypoxia inducible factor-1alpha, through a pathway involving phosphoinositide 3-kinase and the mammalian target of rapamycin. Blood (2002) 100:3767–75. 10.1182/blood-2002-01-0109 12393646

[47] Ly C Arechiga AF Melo JV Walsh CM Ong ST Bcr-Abl kinase modulates the translation regulators ribosomal protein S6 and 4E-BP1 in chronic myelogenous leukemia cells via the mammalian target of rapamycin. Cancer Res (2003) 63:5716–22. 14522890

[48] Carayol N Vakana E Sassano A Kaur S Goussetis DJ Glaser H Critical roles for mTORC2- and rapamycin-insensitive mTORC1-complexes in growth and survival of BCR-ABL-expressing leukemic cells. Proc Natl Acad Sci U S A (2010) 107:12469–74. 10.1073/pnas.1005114107 PMC2906574 20616057

[49] Li J Xue L Hao H Han Y Yang J Luo J Rapamycin provides a therapeutic option through inhibition of mTOR signaling in chronic myelogenous leukemia. Oncol Rep (2012) 27:461–6. 10.3892/or.2011.1502 21993902

[50] Li J Xue L Hao H Li R Luo J Rapamycin combined with celecoxib enhanced antitumor effects of mono treatment on chronic myelogenous leukemia cells through downregulating mTOR pathway. Tumour Biol (2014) 35:6467–74. 10.1007/s13277-014-1820-5 24682932

[51] Mohi MG Boulton C Gu TL Sternberg DW Neuberg D Griffin JD Combination of rapamycin and protein tyrosine kinase (PTK) inhibitors for the treatment of leukemias caused by oncogenic PTKs. Proc Natl Acad Sci U S A (2004) 101:3130–5. 10.1073/pnas.0400063101 PMC365755 14976243

[52] Alves R Goncalves AC Jorge J Alves J Alves da Silva A Freitas-Tavares P Everolimus in combination with Imatinib overcomes resistance in Chronic myeloid leukaemia. Med Oncol (2019) 36:30. 10.1007/s12032-019-1253-5 30796703

[53] Palumbo GA Stella S Pennisi MS Pirosa C Fermo E Fabris S The Role of New Technologies in Myeloproliferative Neoplasms. Front Oncol (2019) 9:321. 10.3389/fonc.2019.00321 31106152 PMC6498877

[54] Bartalucci N Tozzi L Bogani C Martinelli S Rotunno G Villeval JL Co-targeting the PI3K/mTOR and JAK2 signalling pathways produces synergistic activity against myeloproliferative neoplasms. J Cell Mol Med (2013) 17:1385–96. 10.1111/jcmm.12162 PMC4117551 24237791

[55] Guglielmelli P Barosi G Rambaldi A Marchioli R Masciulli A Tozzi L Safety and efficacy of everolimus, a mTOR inhibitor, as single agent in a phase 1/2 study in patients with myelofibrosis. Blood (2011) 118:2069–76. 10.1182/blood-2011-01-330563 PMC3365876 21725052

[56] Bogani C Bartalucci N Martinelli S Tozzi L Guglielmelli P Bosi A mTOR inhibitors alone and in combination with JAK2 inhibitors effectively inhibit cells of myeloproliferative neoplasms. PLoS One (2013) 8:e54826. 10.1371/journal.pone.0054826 23382981 PMC3561413

[57] Vannucchi AM Bogani C Bartalucci N Tozzi L Martinelli S Guglielmelli P Inhibitors of PI3K/Akt and/or mTOR Inhibit the Growth of Cells of Myeloproliferative Neoplasms and Synergize with JAK2 Inhibitor and Interferon. Blood (2011) 118:1638–9. 10.1182/blood.V118.21.3835.3835

[58] Bartalucci N Guglielmelli P Vannucchi AM Rationale for targeting the PI3K/Akt/mTOR pathway in myeloproliferative neoplasms. Clin Lymphoma Myeloma Leuk (2013) 13 Suppl 2:S307–309. 10.1016/j.clml.2013.07.011 24290217

[59] Dutton A Reynolds GM Dawson CW Young LS Murray PG Constitutive activation of phosphatidyl-inositide 3 kinase contributes to the survival of Hodgkin’s lymphoma cells through a mechanism involving Akt kinase and mTOR. J Pathol (2005) 205:498–506. 10.1002/path.1725 15714459

[60] Mark A Hajdu M Varadi Z Sticz TB Nagy N Csomor J Characteristic mTOR activity in Hodgkin-lymphomas offers a potential therapeutic target in high risk disease–a combined tissue microarray, in vitro and in vivo study. BMC Cancer (2013) 13:250. 10.1186/1471-2407-13-250 23693095 PMC3665449

[61] Uddin S Hussain AR Siraj AK Manogaran PS Al-Jomah NA Moorji A Role of phosphatidylinositol 3’-kinase/AKT pathway in diffuse large B-cell lymphoma survival. Blood (2006) 108:4178–86. 10.1182/blood-2006-04-016907 16946303

[62] Hasselblom S Hansson U Olsson M Toren L Bergstrom A Nilsson-Ehle H High immunohistochemical expression of p-AKT predicts inferior survival in patients with diffuse large B-cell lymphoma treated with immunochemotherapy. Br J Haematol (2010) 149:560–8. 10.1111/j.1365-2141.2010.08123.x 20201946

[63] Zhang J Grubor V Love CL Banerjee A Richards KL Mieczkowski PA Genetic heterogeneity of diffuse large B-cell lymphoma. Proc Natl Acad Sci U S A (2013) 110:1398–403. 10.1073/pnas.1205299110 PMC3557051 23292937

[64] Ezell SA Wang S Bihani T Lai Z Grosskurth SE Tepsuporn S Differential regulation of mTOR signaling determines sensitivity to AKT inhibition in diffuse large B cell lymphoma. Oncotarget (2016) 7:9163–74. 10.18632/oncotarget.7036 PMC4891033 26824321

[65] Dal Col J Zancai P Terrin L Guidoboni M Ponzoni M Pavan A Distinct functional significance of Akt and mTOR constitutive activation in mantle cell lymphoma. Blood (2008) 111:5142–51. 10.1182/blood-2007-07-103481 18339899

[66] Calimeri T Ferreri AJM m-TOR inhibitors and their potential role in haematological malignancies. Br J Haematol (2017) 177:684–702. 10.1111/bjh.14529 28146265

[67] Hoppe RT Advani RH Ai WZ Ambinder RF Aoun P Bello CM Hodgkin Lymphoma Version 1.2017, NCCN Clinical Practice Guidelines in Oncology. J Natl Compr Canc Netw (2017) 15:608–38. 10.6004/jnccn.2017.0064 28476741

[68] Johnston PB Pinter-Brown L Rogerio J Warsi G Chau Q Ramchandren R Everolimus for Relapsed/Refractory Classical Hodgkin Lymphoma: Multicenter, Open-Label, Single-Arm, Phase 2 Study. Blood (2012) 120:2740. 10.1182/blood.V120.21.2740.2740

[69] Johnston PB Inwards DJ Colgan JP Laplant BR Kabat BF Habermann TM A Phase II trial of the oral mTOR inhibitor everolimus in relapsed Hodgkin lymphoma. Am J Hematol (2010) 85:320–4. 10.1002/ajh.21664 PMC4420736 20229590

[70] Rocha T Fortier SC Fischer T Perini GF Gaiolla RD Fogliatto L Everolimus as a single agent in refractory or relapsed Hodgkin’s lymphoma: the Brazilian Named Patient Program Experience. Rev Bras Hematol Hemoter (2017) 39:216–22. 10.1016/j.bjhh.2017.03.008 PMC5567422 28830600

[71] Johnston PB Pinter-Brown LC Warsi G White K Ramchandren R Phase 2 study of everolimus for relapsed or refractory classical Hodgkin lymphoma. Exp Hematol Oncol (2018) 7:12. 10.1186/s40164-018-0103-z 29774169 PMC5948762

[72] Witzig TE Reeder CB LaPlant BR Gupta M Johnston PB Micallef IN A phase II trial of the oral mTOR inhibitor everolimus in relapsed aggressive lymphoma. Leukemia (2011) 25:341–7. 10.1038/leu.2010.226 PMC3049870 21135857

[73] Bennani NN LaPlant BR Ansell SM Habermann TM Inwards DJ Micallef IN Efficacy of the oral mTORC1 inhibitor everolimus in relapsed or refractory indolent lymphoma. Am J Hematol (2017) 92:448–53. 10.1002/ajh.24671 28211162

[74] Renner C Zinzani PL Gressin R Klingbiel D Dietrich PY Hitz F A multicenter phase II trial (SAKK 36/06) of single-agent everolimus (RAD001) in patients with relapsed or refractory mantle cell lymphoma. Haematologica (2012) 97:1085–91. 10.3324/haematol.2011.053173 PMC3396682 22315486

[75] Conconi A Raderer M Franceschetti S Devizzi L Ferreri AJ Magagnoli M Clinical activity of everolimus in relapsed/refractory marginal zone B-cell lymphomas: results of a phase II study of the International Extranodal Lymphoma Study Group. Br J Haematol (2014) 166:69–76. 10.1111/bjh.12845 24673512

[76] Padrnos L Ernst B Dueck AC Kosiorek HE Ginos BF Toro A A Novel Combination of the mTORC1 Inhibitor Everolimus and the Immunomodulatory Drug Lenalidomide Produces Durable Responses in Patients With Heavily Pretreated Relapsed Lymphoma. Clin Lymphoma Myeloma Leuk (2018) 18:664–72.e662. 10.1016/j.clml.2018.06.013 30104176

[77] Janku F Oki Y Falchook GS Subbiah V Naing A Bravo VMV Activity of the mTOR inhibitor sirolimus and HDAC inhibitor vorinostat in heavily pretreated refractory Hodgkin lymphoma patients. J Clin Oncol (2014) 32:8508. 10.1200/jco.2014.32.15_suppl.8508

[78] Janku F Garrido-Laguna I Velez-Bravo V Falchook GS Subbiah V Hong DS Significant Activity Of The mTOR Inhibitor Sirolimus and HDAC Inhibitor Vorinostat In Heavily Pretreated Refractory Hodgkin Lymphoma Patients. Blood (2013) 122:3048. 10.1182/blood.V122.21.3048.3048

[79] Witzig TE Geyer SM Ghobrial I Inwards DJ Fonseca R Kurtin P Phase II trial of single-agent temsirolimus (CCI-779) for relapsed mantle cell lymphoma. J Clin Oncol (2005) 23:5347–56. 10.1200/JCO.2005.13.466 15983389

[80] Ansell SM Inwards DJ Rowland KM Jr. Flynn PJ Morton RF Moore DF Jr. Low-dose, single-agent temsirolimus for relapsed mantle cell lymphoma: a phase 2 trial in the North Central Cancer Treatment Group. Cancer (2008) 113:508–14. 10.1002/cncr.23580 PMC3627208 18543327

[81] Tessoulin B Bouabdallah K Burroni B Lamy T Gressin R Cartron G Safety and efficacy of temsirolimus in combination with three different immuno-chemotherapy regimens in relapse and refractory mantle cell lymphoma, final results of the T(3) phase IB trial of the LYSA. Ann Hematol (2020) 99:1771–8. 10.1007/s00277-020-04159-3 32601796

[82] Fenske TS Shah NM Kim KM Saha S Zhang C Baim AE A phase 2 study of weekly temsirolimus and bortezomib for relapsed or refractory B-cell non-Hodgkin lymphoma: A Wisconsin Oncology Network study. Cancer (2015) 121:3465–71. 10.1002/cncr.29502 26079295

[83] Smith SM van Besien K Karrison T Dancey J McLaughlin P Younes A Temsirolimus has activity in non-mantle cell non-Hodgkin’s lymphoma subtypes: The University of Chicago phase II consortium. J Clin Oncol (2010) 28:4740–6. 10.1200/JCO.2010.29.2813 PMC3020703 20837940

[84] Sun SY Rosenberg LM Wang X Zhou Z Yue P Fu H Activation of Akt and eIF4E survival pathways by rapamycin-mediated mammalian target of rapamycin inhibition. Cancer Res (2005) 65:7052–8. 10.1158/0008-5472.CAN-05-0917 16103051

[85] Decker T Hipp S Ringshausen I Bogner C Oelsner M Schneller F Rapamycin-induced G(1) arrest in cycling B-CLL cells is associated with reduced expression of cyclin D3, cyclin E, cyclin A, and survivin. Blood (2003) 101:278–85. 10.1182/blood-2002-01-0189 12393642

[86] Panwalkar A Verstovsek S Giles FJ Mammalian target of rapamycin inhibition as therapy for hematologic malignancies. Cancer (2004) 100:657–66. 10.1002/cncr.20026 14770419

[87] Ghobrial IM Gertz M Laplant B Camoriano J Hayman S Lacy M Phase II trial of the oral mammalian target of rapamycin inhibitor everolimus in relapsed or refractory Waldenstrom macroglobulinemia. J Clin Oncol (2010) 28:1408–14. 10.1200/JCO.2009.24.0994 PMC2834498 20142598

[88] Wang F Zhang W Guo L Bao W Jin N Liu R Gambogic acid suppresses hypoxia-induced hypoxia-inducible factor-1alpha/vascular endothelial growth factor expression via inhibiting phosphatidylinositol 3-kinase/Akt/mammalian target protein of rapamycin pathway in multiple myeloma cells. Cancer Sci (2014) 105:1063–70. 10.1111/cas.12458 PMC4317858 24890366

[89] Adachi M Hoshino Y Izumi Y Sakai H Takagi S Effects of inhibitors of vascular endothelial growth factor receptor 2 and downstream pathways of receptor tyrosine kinases involving phosphatidylinositol 3-kinase/Akt/mammalian target of rapamycin or mitogen-activated protein kinase in canine hemangiosarcoma cell lines. Can J Vet Res (2016) 80:209–16. PMC4924555 27408334

[90] Shi Y Gera J Hu L Hsu JH Bookstein R Li W Enhanced sensitivity of multiple myeloma cells containing PTEN mutations to CCI-779. Cancer Res (2002) 62:5027–34. 12208757

[91] Chen JQ Ying YL Zhu HJ Zhu TJ Qu CS Jiang JH Curcumin-induced promoter hypermethylation of the mammalian target of rapamycin gene in multiple myeloma cells. Oncol Lett (2019) 17:1108–14. 10.3892/ol.2018.9662 PMC6312997 30655872

[92] Lamanuzzi A Saltarella I Desantis V Frassanito MA Leone P Racanelli V Inhibition of mTOR complex 2 restrains tumor angiogenesis in multiple myeloma. Oncotarget (2018) 9:20563–77. 10.18632/oncotarget.25003 PMC5945497 29755672

[93] Jin HG Wu GZ Wu GH Bao YG Combining the mammalian target of rapamycin inhibitor, rapamycin, with resveratrol has a synergistic effect in multiple myeloma. Oncol Lett (2018) 15:6257–64. 10.3892/ol.2018.8178 PMC5920858 29731844

[94] Li J Liu ZY Li YQ Jing Q Wang HL Liu H Everolimus shows synergistic antimyeloma effects with bortezomib via the AKT/mTOR pathway. J Investig Med (2019) 67:39-47 10.1136/jim-2018-000780 29997148

[95] Li J Liu ZY Li YQ Jing Q Wang HL Liu H Everolimus shows synergistic antimyeloma effects with bortezomib via the AKT/mTOR pathway. J Invest Med (2019) 67:39–47. 10.1136/jim-2018-000780 29997148

[96] Gunther A Baumann P Burger R Kellner C Klapper W Schmidmaier R Activity of everolimus (RAD001) in relapsed and/or refractory multiple myeloma: a phase I study. Haematologica (2015) 100:541–7. 10.3324/haematol.2014.116269 PMC4380728 25682600

[97] Yee AJ Hari P Marcheselli R Mahindra AK Cirstea DD Scullen TA Outcomes in patients with relapsed or refractory multiple myeloma in a phase I study of everolimus in combination with lenalidomide. Br J Haematol (2014) 166:401–9. 10.1111/bjh.12909 24761838

[98] Hofmeister CC Yang X Pichiorri F Chen P Rozewski DM Johnson AJ Phase I trial of lenalidomide and CCI-779 in patients with relapsed multiple myeloma: evidence for lenalidomide-CCI-779 interaction via P-glycoprotein. J Clin Oncol (2011) 29:3427–34. 10.1200/JCO.2010.32.4962 PMC3164245 21825263

[99] Ghobrial IM Weller E Vij R Munshi NC Banwait R Bagshaw M Weekly bortezomib in combination with temsirolimus in relapsed or relapsed and refractory multiple myeloma: a multicentre, phase 1/2, open-label, dose-escalation study. Lancet Oncol (2011) 12:263–72. 10.1016/S1470-2045(11)70028-6 21345726

[100] Wang CY Ma S Bi SJ Su L Huang SY Miao JY Enhancing autophagy protects platelets in immune thrombocytopenia patients. Ann Transl Med (2019) 7:134. 10.21037/atm.2019.03.04 31157255 PMC6511561

[101] Li J Wang Z Dai L Cao L Su J Zhu M Effects of rapamycin combined with low dose prednisone in patients with chronic immune thrombocytopenia. Clin Dev Immunol (2013) 2013:548085. 10.1155/2013/548085 24363761 PMC3865723

[102] Bride KL Vincent T Smith-Whitley K Lambert MP Bleesing JJ Seif AE Sirolimus is effective in relapsed/refractory autoimmune cytopenias: results of a prospective multi-institutional trial. Blood (2016) 127:17–28. 10.1182/blood-2015-07-657981 26504182 PMC4705607

[103] Jasinski S Weinblatt ME Glasser CL Sirolimus as an Effective Agent in the Treatment of Immune Thrombocytopenia (ITP) and Evans Syndrome (ES): A Single Institution’s Experience. J Pediatr Hematol Oncol (2017) 39:420–4. 10.1097/MPH.0000000000000818 28267088

[104] Miano M Rotulo GA Palmisani E Giaimo M Fioredda F Pierri F Sirolimus as a rescue therapy in children with immune thrombocytopenia refractory to mycophenolate mofetil. Am J Hematol (2018) 93:E175–7. 10.1002/ajh.25119 29675829

[105] Feng YM Xiao YS Yan HJ Wang P Zhu W Cassady K Sirolimus as Rescue Therapy for Refractory/Relapsed Immune Thrombocytopenia: Results of a Single-Center, Prospective, Single-Arm Study. Front Med (2020) 7:110. 10.3389/fmed.2020.00110 PMC7136762 32296709

[106] Neely J von Scheven E Autoimmune haemolytic anaemia and autoimmune thrombocytopenia in childhood-onset systemic lupus erythematosus: updates on pathogenesis and treatment. Curr Opin Rheumatol (2018) 30:498–505. 10.1097/BOR.0000000000000523 29979258

[107] Park JA Lee HH Kwon HS Baik CR Song SA Lee JN Sirolimus for Refractory Autoimmune Hemolytic Anemia after Allogeneic Hematopoietic Stem Cell Transplantation: A Case Report and Literature Review of the Treatment of Post-Transplant Autoimmune Hemolytic Anemia. Transfus Med Rev (2016) 30:6–14. 10.1016/j.tmrv.2015.09.001 26481836

[108] Salama A Treatment Options for Primary Autoimmune Hemolytic Anemia: A Short Comprehensive Review. Transfus Med Hemother (2015) 42:294–301. 10.1159/000438731 26696797 PMC4678315

[109] Mqadmi A Zheng XY Yazdanbakhsh K CD4(+) CD25(+) regulatory T cells control induction of autoimmune hemolytic anemia. Blood (2005) 105:3746–8. 10.1182/blood-2004-12-4692 PMC1895013 15637139

[110] Richards AL Kapp LM Wang X Howie HL Hudson KE Regulatory T Cells Are Dispensable for Tolerance to RBC Antigens. Front Immunol (2016) 7:348. 10.3389/fimmu.2016.00348 27698653 PMC5027202

[111] Kamesaki T Molecular mechanisms of autoimmune hemolytic anemia. Rinsho Ketsueki (2015) 56:846–54. 10.11406/rinketsu.56.846 26251148

[112] Miano M How I manage Evans Syndrome and AIHA cases in children. Br J Haematol (2016) 172:524–34. 10.1111/bjh.13866 26625877

[113] Acquazzino MA Fischer RT Langnas A Coulter DW Refractory autoimmune hemolytic anemia after intestinal transplant responding to conversion from a calcineurin to mTOR inhibitor. Pediatr Transplant (2013) 17:466–71. 10.1111/petr.12101 23730873

[114] Kalfa TA Warm antibody autoimmune hemolytic anemia. Hematol Am Soc Hematol Educ Program (2016) 2016:690–7. 10.1182/asheducation-2016.1.690 PMC6142448 27913548

[115] Yan H Wang P Quan Y Gao L Liu Y Zhang C Clinical efficacy of sirolimus in treatment of relapsed and refractory autoimmune hemolytic anemia in adults. J Third Mil Med Univ (2020) 42:1757–62. 10.16016/j.1000-5404.202005102

[116] Li P Huang P Yang Y Hao M Peng H Li F Updated Understanding of Autoimmune Lymphoproliferative Syndrome (ALPS). Clin Rev Allergy Immunol (2016) 50:55–63. 10.1007/s12016-015-8466-y 25663566

[117] Cooling LL Sherbeck J Mowers JC Hugan SL Development of red blood cell autoantibodies following treatment with checkpoint inhibitors: a new class of anti-neoplastic, immunotherapeutic agents associated with immune dysregulation. Immunohematology (2017) 33:15–21. 28425751

[118] Gu H Wu RH Advance of Mechanisms and Clinical Applications about Rapamycin for Treating Immune Mediated Hemocytopenia. Zhongguo Shi Yan Xue Ye Xue Za Zhi (2018) 26:1836–40. 10.7534/j.issn.1009-2137.2018.06.043 30501730

[119] Volkl S Rensing-Ehl A Allgauer A Schreiner E Lorenz MR Rohr J Hyperactive mTOR pathway promotes lymphoproliferation and abnormal differentiation in autoimmune lymphoproliferative syndrome. Blood (2016) 128:227–38. 10.1182/blood-2015-11-685024 27099149

[120] Teachey DT Obzut DA Axsom K Choi JK Goldsmith KC Hall J Rapamycin improves lymphoproliferative disease in murine autoimmune lymphoproliferative syndrome (ALPS). Blood (2006) 108:1965–71. 10.1182/blood-2006-01-010124 PMC1895548 16757690

[121] Teachey DT Seif AE Grupp SA Advances in the management and understanding of autoimmune lymphoproliferative syndrome (ALPS). Br J Haematol (2010) 148:205–16. 10.1111/j.1365-2141.2009.07991.x PMC2929682 19930184

[122] George LA Teachey DT Optimal Management of Autoimmune Lymphoproliferative Syndrome in Children. Pediatr Drugs (2016) 18:261–72. 10.1007/s40272-016-0175-3 PMC9677085 27139496

[123] Teachey DT Greiner R Seif A Attiyeh E Bleesing J Choi J Treatment with sirolimus results in complete responses in patients with autoimmune lymphoproliferative syndrome. Br J Haematol (2009) 145:101–6. 10.1111/j.1365-2141.2009.07595.x PMC2819393 19208097

[124] Dragana JM Dimitrije BC Srda JJ Lidija DB Nada KR Nada KKJ Rapid Regression of Lymphadenopathy upon Rapamycin Treatment in a Child With Autoimmune Lymphoproliferative Syndrome. Pediatr Blood Cancer (2009) 53:1117–9. 10.1002/pbc.22151 19588524

[125] Miano M Scalzone M Perri K Palmisani E Caviglia I Micalizzi C Mycophenolate mofetil and Sirolimus as second or further line treatment in children with chronic refractory Primitive or Secondary Autoimmune Cytopenias: a single centre experience. Br J Haematol (2015) 171:247–53. 10.1111/bjh.13533 26058843

[126] Arenas DJ Floess K Kobrin D Pai RL Srkalovic MB Tamakloe MA Increased mTOR activation in idiopathic multicentric Castleman disease. Blood (2020) 135:1673–84. 10.1182/blood.2019002792 PMC7205815 32206779

[127] Medinger M Drexler B Lengerke C Passweg J Pathogenesis of Acquired Aplastic Anemia and the Role of the Bone Marrow Microenvironment. Front Oncol (2018) 8:587. 10.3389/fonc.2018.00587 30568919 PMC6290278

[128] Young NS Current concepts in the pathophysiology and treatment of aplastic anemia. Hematol-Am Soc Hematol Educ Program (2013) 2013(1):76–81. 10.1182/asheducation-2013.1.76 PMC6610029 24319166

[129] Gill H Wong RSM Kwong YL From chronic immune thrombocytopenia to severe aplastic anemia: recent insights into the evolution of eltrombopag. Ther Adv Hematol (2017) 8:159–74. 10.1177/2040620717693573 PMC5407506 28473904

[130] Boddu PC Kadia TM Updates on the pathophysiology and treatment of aplastic anemia: a comprehensive review. Expert Rev Hematol (2017) 10:433–48. 10.1080/17474086.2017.1313700 28452257

[131] Liu SL Zhou YM Tang DB Zhou N Zheng WW Tang ZH Rapamycin ameliorates immune-mediated aplastic anemia by inhibiting the proliferation and metabolism of T cells. Biochem Biophys Res Commun (2019) 518:212–8. 10.1016/j.bbrc.2019.08.034 31434610

[132] Wang X Ma FX Lu SH Chi Y Chen F Li X Effects of rapamycin on biological characteristics of bone marrow mesenchymal stem cells from patients with aplastic anemia. Zhongguo Shi Yan Xue Ye Xue Za Zhi (2014) 22:762–6. 10.7534/j.issn.1009-2137.2014.03.035 24989291

[133] He G Zhang X Wu D Sun A Wang X Relapse of aplastic anemia responsive to sirolimus combined with cyclosporine. Pediatr Blood Cancer (2011) 56:1133–5. 10.1002/pbc.22865 21488160

[134] Scheinberg P Wu CO Nunez O Scheinberg P Boss C Sloand EM Treatment of severe aplastic anemia with a combination of horse antithymocyte globulin and cyclosporine, with or without sirolimus: a prospective randomized study. Haematologica (2009) 94:348–54. 10.3324/haematol.13829 PMC2649367 19181786

[135] Yang C Chen FF Long ZB Du YL Li HM Chen M Effect of sirolimus on erythropoiesis of K562 cell line and patients with pure red cell aplasia in vitro. Zhonghua Xue Ye Xue Za Zhi (2018) 39:310–3. 10.3760/cma.j.issn.0253-2727.2018.04.011 PMC7342129 29779328

[136] Ovilla R Succesful treatment with Sirolimus in a refractory case of pure red cell aplasia (PRCA). Blood (2007) 110:11b–b. 10.1182/blood.V110.11.3778.3778

[137] Jiang H Zhang H Wang Y Qi W Cao Q Xing L Sirolimus for the treatment of multi-resistant pure red cell aplasia. Br J Haematol (2019) 184:1055–8. 10.1111/bjh.15245 29741762

[138] Long ZB Yu F Du YL Li HM Chen M Zhuang JL Successful treatment of refractory/relapsed acquired pure red cell aplasia with sirolimus. Ann Hematol (2018) 97:2047–54. 10.1007/s00277-018-3431-5 29982851

[139] Ferrara JLM Levine JE Reddy P Holler E Graft-versus-host disease. Lancet (2009) 373:1550–61. 10.1016/S0140-6736(09)60237-3 PMC2735047 19282026

[140] Ghimire S Weber D Mavin E Wang XN Dickinson AM Holler E Pathophysiology of GvHD and Other HSCT-Related Major Complications. Front Immunol (2017) 8:79. 10.3389/fimmu.2017.00079 28373870 PMC5357769

[141] Coenen JJ Koenen HJ van Rijssen E Kasran A Boon L Hilbrands LB Rapamycin, not cyclosporine, permits thymic generation and peripheral preservation of CD4+ CD25+ FoxP3+ T cells. Bone Marrow Transplant (2007) 39:537–45. 10.1038/sj.bmt.1705628 17351648

[142] Armand P Gannamaneni S Kim HT Cutler CS Ho VT Koreth J Improved survival in lymphoma patients receiving sirolimus for graft-versus-host disease prophylaxis after allogeneic hematopoietic stem-cell transplantation with reduced-intensity conditioning. J Clin Oncol (2008) 26:5767–74. 10.1200/JCO.2008.17.7279 PMC2645102 19001324

[143] Armand P Kim HT Sainvil MM Lange PB Giardino AA Bachanova V The addition of sirolimus to the graft-versus-host disease prophylaxis regimen in reduced intensity allogeneic stem cell transplantation for lymphoma: a multicentre randomized trial. Br J Haematol (2016) 173:96–104. 10.1111/bjh.13931 26729448 PMC4809783

[144] Sandmaier BM Kornblit B Storer BE Olesen G Maris MB Langston AA Addition of sirolimus to standard cyclosporine plus mycophenolate mofetil-based graft-versus-host disease prophylaxis for patients after unrelated non-myeloablative haemopoietic stem cell transplantation: a multicentre, randomised, phase 3 trial. Lancet Haematol (2019) 6:e409–18. 10.1016/S2352-3026(19)30088-2 PMC6686903 31248843

[145] Wang L Gu Z Zhai R Li D Zhao S Luo L The efficacy and safety of sirolimus-based graft-versus-host disease prophylaxis in patients undergoing allogeneic hematopoietic stem cell transplantation: a meta-analysis of randomized controlled trials. Transfusion (Paris) (2015) 55:2134–41. 10.1111/trf.13110 25857725

[146] Pidala J Hamadani M Dawson P Martens M Alousi AM Jagasia M Randomized multicenter trial of sirolimus vs prednisone as initial therapy for standard-risk acute GVHD: the BMT CTN 1501 trial. Blood (2020) 135:97–107. 10.1182/blood.2019003125 31738834 PMC6952830

[147] Carpenter PA Logan BR Lee SJ Weisdorf DJ Johnston L Costa LJ A phase II/III randomized, multicenter trial of prednisone/sirolimus versus prednisone/sirolimus/calcineurin inhibitor for the treatment of chronic graft-versus-host disease: BMT CTN 0801. Haematologica (2018) 103:1915–24. 10.3324/haematol.2018.195123 PMC6278959 29954931

[148] Zhu W Feng YM Chen T Yao H Quan Y Rao J The clinical observation of sirolimus combined with calcineurin inhibitors for steroid-resistant/steroid-dependent extensive cGVHD. Zhonghua Xue Ye Xue Za Zhi (2020) 41:716–22. 10.3760/cma.j.issn.0253-2727.2020.09.003 PMC7595869 33113602

